# Pressure pain thresholds in individuals with knee pain: a cross-sectional study

**DOI:** 10.1186/s12891-021-04408-0

**Published:** 2021-06-05

**Authors:** Charlotte Sylwander, Ingrid Larsson, Emma Haglund, Stefan Bergman, Maria L.E. Andersson

**Affiliations:** 1 School of Health and Welfare, Halmstad University, Halmstad, Sweden; 2 Spenshult Research and Development Centre, Bäckagårdsvägen 47, SE-302 74 Halmstad, Sweden; 3 Department of Clinical Sciences, Section of Rheumatology, Lund University, Lund, Sweden; 4 Rydberg Laboratory of Applied Sciences, Halmstad University, Halmstad, Sweden; 5 Primary Care, School of Public Health and Community Medicine, Institute of Medicine, The Sahlgrenska Academy at University of Gothenburg, Gothenburg, Sweden

**Keywords:** Pain sensitivity, Pressure pain thresholds, Knee osteoarthritis, Chronic widespread pain, Obesity, Overweight

## Abstract

**Background:**

Knee osteoarthritis (KOA), chronic widespread pain (CWP) and overweight/obesity are public health problems that often coincide, and there is a multifactorial and unclear relationship between them. The study aimed to (1) investigate pain sensitivity, assessed by pressure pain thresholds (PPTs), among women and men with knee pain and (2) associations with, respectively, radiographic KOA (rKOA), CWP, and overweight/obesity.

**Methods:**

Baseline data from an ongoing longitudinal study involving 280 individuals with knee pain in the 30–60 age group. Pain sensitivity was assessed by PPTs on eight different tender points using a pressure algometer. The participants’ knees were x-rayed. Self-reported CWP and number of pain sites were assessed with a pain figure, and overweight/obesity was measured using body mass index (BMI), visceral fat area (VFA), and body fat percentage, assessed with a bioimpedance. Associations were analysed using regression analyses.

**Results:**

Women reported lower PPTs than men (*p* < 0.001), but no PPTs differences were found between those with and without rKOA. Low PPTs was associated with female sex, more pain sites, CWP, and a higher VFA and body fat percentage. The tender points second rib and the knees were most affected. The prevalence of CWP was 38 %.

**Conclusions:**

The modifiable factors, increased VFA, and body fat could be associated with increased pain sensitivity among individuals with knee pain. Longitudinal studies are needed to further investigate the associations.

**Supplementary Information:**

The online version contains supplementary material available at 10.1186/s12891-021-04408-0.

## Introduction

Knee osteoarthritis (KOA) is a common disease in the general population, and the prevalence is up to 14 % among uninjured adults under the age of 40 years and increases with age (40 or older) to 19–43 % [1]. The prevalence has increased during recent years [2]. KOA affects the joint capsule, the articular cartilage, and cartilaginous bones and ligaments, causing disability and pain [3]. Pain is the symptom in KOA that causes most disability [4]. Individuals with KOA may have central sensitisation of the nociceptive system reporting low pressure pain thresholds (PPTs) in both the affected knee (peripheral sensitisation) and remote sites (central sensitisation) [5, 6]. Increased pain sensitivity (lower PPTs) has been reported as a premorbid risk factor for worsening KOA symptoms and pain conditions [7, 8]. Pain sensitivity has been suggested to be more associated with the severity of symptoms rather than radiographic severity, but the mechanisms behind are unknown [5, 9, 10]. However, not all individuals with KOA experience problems with pain [11, 12], and the association between KOA and pain is still unclear [7].

A majority of individuals with knee pain develop KOA [13], and approximately 30 % of individuals with knee pain develop chronic widespread pain (CWP) regardless of having KOA or not [14]. This prevalence is higher than in the general population, where the prevalence for CWP is 10 % [15]. In Europe, pain is one of the top reasons to seek medical care [16], and an estimated one-third of the adult population lives with chronic pain [17], usually defined as pain for three months or more [18]. A subgroup of those with chronic pain reports CWP [19], and central sensitisation could induce the spread of the pain [20]. CWP and higher pain sensitivity are more frequent in women than men [21].

Reports of chronic pain are 20 % higher among overweight (25-29.9) individuals, compared to normal-weight individuals; for obese (BMI 30-34.9) and morbidly obese (BMI ≥ 40), the increase is up to 68 and 254 %, respectively, compared to normal-weighted [22]. Increased body mass is also a risk factor for developing KOA [23] and is associated with osteoarthritis progression and severity [22]. Among overweight and obese individuals, depending on body site, the results regarding pain sensitivity are conflicting, but, overall, research tends to support increased pain sensitivity [12, 24].

KOA, chronic pain and overweight/obesity often coincide, and there is a multifactorial and unclear relationship between them. Individuals with knee pain have a higher risk of developing both KOA and CWP, and overweight/obesity could be a modifiable inducing risk factor. It is also of interest to see if high pain sensitivity could be an early indicator of developing a chronic pain state.

The study aimed to (1) investigate pain sensitivity, assessed by PPT, among women and men with knee pain and (2) associations with, respectively, radiographic KOA, CWP, and overweight/obesity.

## Method

### Study design

This was a cross-sectional study based on baseline data in an ongoing longitudinal cohort study including 301 individuals with knee pain in the southwest of Sweden. The participants were recruited: (1) by primary health care clinics when searching care for knee pain, and (2) by way of advertisements in local newspapers. The inclusion criteria were: current knee pain, aged 30–60 years, with no former diagnosed radiographic KOA (rKOA), and no rheumatologic disorder or cruciate ligament injury. Enrolments took place from 2017 to 2019. A general practitioner examined all participants to confirm the exclusion of rheumatologic disorder. The participants also completed a questionnaire, including pain distribution, socio-demographics, self-reported fibromyalgia, and the Knee injury and Osteoarthritis Outcome Score (KOOS).

### Participants

Out of the 301 participants, 280 (71 % women; median age 53, IQR 47–58) participated in the PPT measurement at baseline. The missing data (n = 21) were mainly due to temporary technical problems with the PPT algometer, and one participant decided not to complete the measurement.

### Outcome Measures

The main outcome was pain sensitivity, assessed by PPT. Other outcome measures were rKOA, CWP, and overweight/obesity. PPT and overweight/obesity were assessed during a clinical examination, and PPTs were measured with a digital pressure algometer. The use of a digital pressure algometer to measure PPT has demonstrated good validity and reliability in individuals, both with [25, 26] and without rKOA [27].

### Pressure pain thresholds

The PPTs were measured on eight predefined tender points out of the 18 points as part of the definition of fibromyalgia [19]. The locations of the eight tender points were: trapezius (bilateral, midpoint of the upper border); second rib (right side, at the second costochondral junctions, just lateral to the junctions on the upper surfaces); lateral epicondyle (right side, 2 cm distal to the epicondyles); knees (bilateral, at the medial fat pad proximal to the joint line); gluteal (bilateral, in upper outer quadrants of the buttocks in the anterior fold of the gluteus maximus muscle). The eight tender points were chosen to enable a reflection of general allodynia and not only a higher pain sensitivity around the knees.

A hand-held pressure algometer with a 1 cm^2^ rubber probe was used, together with a computer interface with an assistant linear response to force application (AlgoMed, Medoc, Ramat Yishai, Israel). A constant rate of force has been shown to have the highest reliability [28]. Two trials were assessed on each tender point, at a minimum of 30 s apart. The pressure gradually increased from 0 to a maximum of 1000 kilopascals (kPa) at a rate of approximately 40 kPa/s, or until the participant pressed the stop button. The participants were informed that the aim of the test was to measure the pain thresholds and not pain tolerance level, and received the following instruction: *“Press the button when you feel the first sensation of pressure shifting to pain”.* The measurement occurred before physical activity or after 30 min of rest [29]. The raters (n = 5, four exercise physiologists and one physiotherapist) had adequate knowledge in anatomy and palpation and had gone through a minimum of 1-hour practice before the measurement [30]. The raters had no relationship with the participants and no knowledge of their pain status.

### Radiographic knee osteoarthritis

The participants’ knees were x-rayed at one hospital and assessed by experienced radiologists, and rKOA was defined according to the Ahlbäck five grading scale [31]. A result of grade 1 or more was considered as rKOA.

### Chronic widespread pain

CWP was defined, in accordance with the American College of Rheumatology’s criteria, as having pain for three months or more, present below and above the waist, on both sides of the body, and in the axial skeleton [19]. Self-reported CWP was assessed by a pain figure with 18 predefined areas (pain sites). According to the criteria, the participants were categorised into three different pain groups: CWP, chronic regional pain (CRP), if the criteria for CWP were not meet, or no chronic pain (NCP) [32].

### Overweight/obesity

Overweight/obesity was assessed by body mass index (BMI, kg/m^2^), visceral fat area (VFA, cm^2^), and body fat percentage (%), which were assessed using a multifrequency bioelectrical impedance analysis (InBody 770®). The Inbody 770 has been tested for validity showing a strong correlation to dual-energy X-ray absorptiometry (DXA*)* [33]. A VFA score of > 100 was considered an increased health risk [34].

### Questionnaire

The questionnaire included: questions about socio-demographics (age, marital status, education level), most painful knee, fibromyalgia (if the participant had been diagnosed with fibromyalgia by a physician), and the Swedish validated KOOS version [35, 36], which was used to describe the sample further. The most painful knee was identified by the questionnaire, the pain figure or from a question during the clinical examination. Some of the participants had fluctuating knee pain and reported, therefore, no knee pain when filling out the questionnaire. KOOS consists of 42 items with a Likert scale, creating five different subscales: Pain, symptom, function in daily living (ADL), function in sport and recreation (Sport/Rec), and knee-related quality of life (QoL) [36]. The scores ranged between 0 and 100, where 0 represents extreme knee problems and 100 no problems, and the minimal clinically important changes suggested for KOOS are 8–10 [37].

### Statistical analysis

Baseline characteristics were socio-demographics, rKOA, pain group (NCP, CRP, and CWP), number of pain sites, comorbidity (fibromyalgia), BMI, VFA, body fat percentage, and the KOOS subscales. The PPT, obesity variables and KOOS subscales showed no normal distribution, whereas nonparametric statistics were used. The results were presented as median with interquartile range (IQR). The mean of the two PPTs on each tender point was used in the analysis, and bilateral sites (trapezius, knees, and gluteus) were combined into one mean-aggregated pain threshold value [38]. Because of the significant differences in median PPT score in all eight tender points between women and men (men had higher PPT, *p* < 0.001), the analyses were stratified for sex, except for the regression analyses to maintain power. Based on differences in KOOS pain between pain groups (CWP and NCP/CRP), a sample size of 188 patients were needed to reach a power of 95 % and an alpha of 0.05 (two-tailed) [14].

A chi-squared test was used to analyse proportions, and the Mann-Whitney U test was used for ordinal and scale data to test the differences between groups. Overweight/obesity was defined by having VFA > 100. Since the PPTs had sufficient linearity, a univariate regression analysis was used to study associations between PPTs and, respectively, rKOA, pain (number of pain sites and CWP) and obesity variables. Variables having a p-value above 0.25 in the univariate analysis were included in the multivariate regression analysis [39] controlled for age and sex. Results were considered significant if *p* ≤ 0.05. All analyses were performed in IBM SPSS 24 statistical package for Windows (released 2016; IBM Corp., Armonk, NY, USA).

### Ethical considerations

All participants signed a written informed consent document. The study adhered to the Helsinki Declaration [40] and was approved by the Swedish Ethics Review Authority (Dnr 2016/816; 2017/205).

## Results

**Fig. 1:**
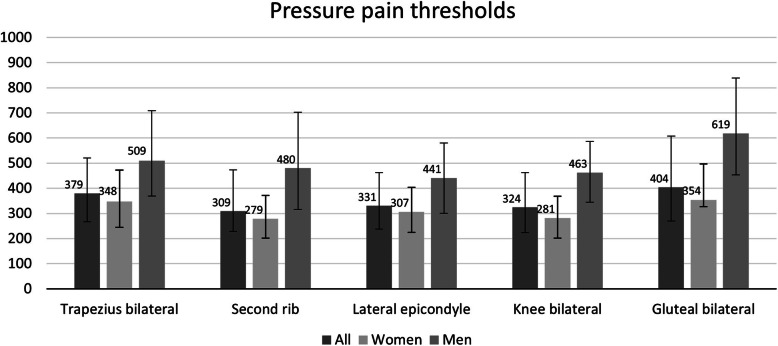
Overview of the eight different pressure pain thresholds s in the whole sample, and separately for women and men, presented as median kilopascals (kPa) with interquartile range (*p* < 0.001). Details can be found in Additional file 1

Out of the 280 participants included, 214 (81 %) were married or cohabiting, and 126 (48 %) had a higher education (university), women 47 % and men 27.5 % (*p* < 0.001). Women reported more pain sites, lower BMI but higher VFA and body fat percentage than men (Table 1). Women also reported lower scores in four out of five KOOS subscales (Pain, Symptom, ADL, Sport/Rec) than men, but only clinically relevant in Sport/Rec (Table 1). Almost a quarter of the participants (24 %) were found to have rKOA. The prevalence of CWP was 38 % in the whole sample; 41 % among women and 30 % among men. Median BMI was 26 (IQR 23–29), indicating that half of the participants were overweight. The median VFA was 103 (IQR 73–145), and 52 % had a high VFA with increasing health risks (Table 1). Women reported lower PPTs than men at all eight tender points (*p* < 0.001) (Fig. 1, details in Additional file 1).

**Table 1:** Descriptive statistics for the whole sample and separately for women (*n* = 199) and men (*n* = 81). BMI, VFA and body fat were assessed during the clinical examination, other from the questionnaire

	Number	All Median (IQR)	Women Median (IQR)	Men Median (IQR)	*p*-value
Age	280	53 (47–58)	54 (47–58)	52 (47–58)	0.824
rKOA^a^, n (%)	268	65 (24)	45 (24)	20 (25)	0.853
Pain group, n (%) NCP CRP CWP	261	20 (8) 142 (54) 99 (38)	14 (8) 95 (51) 76 (41)	6 (8) 47 (62) 23 (30)	0.252
Numbers of pain sites	261	4 (2–7)	5 (2–7)	3 (1–5)	0.003
Painful knee^b^, n (%) No knee pain* Right Left Both	269	28 (10) 57 (21) 47 (18) 137 (51)	19 (10) 42 (22) 29 (15) 102 (53)	9 (12) 15 (19.5) 18 (23) 35 (45.5)	0.376
Fibromyalgia, n (%)	275	8 (3)	7 (4)	1 (1)	0.309
BMI	277	26 (23–29)	25 (23–29)	27 (25–29)	0.019
VFA, cm^2^	275	103 (73–145)	108 (73–155)	93 (73–93)	0.034
VFA > 100 cm^2^, n (%)	275	142 (52)	107 (55)	35 (44)	0.122
Body fat, (%)	275	30 (24–37)	33 (27–39)	23 (19–30)	< 0.001
KOOS (worst-best) Pain Symptom ADL Sport/Rec QoL	255	75 (61–83) 71 (57–82) 85 (75–94) 45 (25–67) 50 (38–63)	75 (58–81) 71 (57–82) 84 (74–93) 40 (25–65) 50 (38–68)	76 (69–89) 75 (61–86) 88 (80–96) 55 (40–70) 56 (44–63)	0.013 0.024 0.030 0.006 0.513

^a^ Having a score ≥ 1 on the Ahlbäck scale for rKOA

^b^ Knee pain status at the questionnaire

*rKOA* radiographic knee osteoarthritis; *NCP* no chronic pain; *CRP* chronic regional pain; *CWP* chronic widespread pain; *BMI* body mass index; *VFA* visceral fat area; *KOOS* Knee injury and Osteoarthritis Outcome Score; *ADL* function in daily living; *Sport/Rec* function in sport and recreation; *QoL* knee-related quality of life

### Pressure pain thresholds

Neither women nor men with and without rKOA showed statistical differences in the PPTs (*p* > 0.05) (Additional file 2). When comparing PPTs and pain distribution, women with CWP reported lower PPTs than women with CRP/NCP (Table 2). No differences between PPT and pain distribution were found among men. Comparing PPTs between overweight/obese (VFA > 100) and normal-weight women (VFA ≤ 100), overweight/obese women reported significant lower PPTs at the second rib and the knees (Table 3). Among overweight/obese men, lower PPTs were reported at the knees, compared to normal-weighted.

**Table 2:** PPTs at the different tender points among women and men between the pain groups: CWP, CRP, NCP. The PPTs were presented as median and interquartile range (IQR)

PPT median kPa (IQR)	Women			Men		
	CWP *n* = 76	CRP/NCP *n* = 109	*p*-value	CWP *n* = 23	CRP/NCP *n* = 53	*p*-value
Trapezius bilateral	302 (215–388)	387 (286–502)	< 0.001	509 (271–803)	513 (404–688)	0.587
s rib	224 (157–325)	317 (251–430)	< 0.001	478 (271–695)	489 (324–707)	0.734
Lateral epicondyle	254 (204–351)	345 (269–452)	< 0.001	412 (286–607)	450 (334–563)	0.923
Knee bilateral	234 (168–318)	325 (234–431)	< 0.001	393 (346–647)	467 (344–583)	0.672
Gluteal bilateral	287 (201–400)	398 (269–532)	< 0.001	643 (431–847)	619 (497–799)	0.739

*CRP* chronic regional pain; *CWP* chronic widespread pain; *NCP* no chronic pain; *PPT* pressure pain thresholds

**Table 3:** PPTs in the different tender points among women and men with VFA over and under 100. PPTs were presented as median and interquartile range (IQR)

PPT median kPa (IQR)	Women			Men		
	VFA > 100 *n* = 107	VFA ≤ 100 *n* = 89	*p*-value	VFA > 100 *n* = 35	VFA ≤ 100 *n* = 44	*p*-value
Trapezius bilateral	348 (244–488)	342 (241–451)	0.710	527 (361–673)	482 (375–784)	0.824
s rib	262 (176–361)	299 (243–396)	0.007	459 (300–690)	493 (329–753)	0.456
Lateral epicondyle	311 (218–406)	305 (228–398)	0.922	393 (274–615)	453 (349–568)	0.295
Knee bilateral	256 (187–344)	307 (222–426)	0.002	383 (324–551)	518 (373–682)	0.034
Gluteal bilateral	325 (225–485)	370 (259–500)	0.159	594 (432–764)	655 (442–840)	0.430

*VFA* visceral fat area; *PPT* pressure pain thresholds

### Associations with lower pressure pain thresholds

According to the univariate regressions, older age was associated with higher PPTs in all tender points except for the lateral epicondyle (Table 4). Being a woman, having a higher number of pain sites, CWP, and a higher body fat percentage were associated with lower PPTs at all tender points. A high VFA was associated with lower PPTs at the second rib and the knee. Having rKOA was not significant associated with higher pain sensitivity at any of the tender points. In the multivariate regression analysis, having CWP and a higher number of pain sites were associated with lower PPTs at all tender points (Table 5). Increased VFA and body fat percentage were associated with lower PPTs at the second rib and the knees.

**Table 4:** Univariate regression analysis in the whole sample of associations between the different PPTs

		Trapezius bilateral		Second rib		Lateral epicondyle		Knee bilateral		Gluteal bilateral	
	n	B (95 % CI)	*p*-value	B (95 % CI)	*p*-value	B (95 % CI)	*p*-value	B (95 % CI)	*p*-value	B (95 % CI)	*p*-value
Age	280	3.83 (1.10–6.56)	0.006	3.01 (0.17–5.84)	0.038	2.07 (-0.31–4.45)	0.088	2.55 (0.09–5.01)	0.043	4.55 (1.37–7.73)	0.005
Sex^a^	280	-184.87 (-233.81– -135.92)	< 0.001	-226.14 (-274.59– -177.68)	< 0.001	-136.25 (-179.68– -92.82)	< 0.001	-183.89 (-226.58– -141.21)	< 0.001	-267.63 (-321.53– -213.73)	< 0.001
rKOA^b^	268	31.09 (-27.24–89.41)	0.296	26.90 (-33.43–87.22)	0.382	36.88 (-13.64– -87.41)	0.152	18.01 (-34.25–70.28)	0.499	24.28 (-43.65–92.21)	0.484
Numbers of pain sites	261	-12.28 (-18.72– -5.81)	< 0.001	-11.05 (-17.75– -4.34)	0.001	-10.27 (-15.88– -4.65)	< 0.001	-12.51 (-18.33– -6.69)	< 0.001	-14.82 (-22.28– -7.35)	< 0.001
Pain group^c^	261	-90.37 (-140.98– -39.76)	< 0.001	-82.82 (-135.21– -30.44)	0.002	-79.99 (-118.95– -31.04)	0.001	-74.82 (-120.88– -28.76)	0.001	-101.19 (159.84– -42.54)	0.001
BMI	277	6.63 (1.62–11.64)	0.010	-1.20 (-6.45–4.05)	0.655	4.03 (-0.33–8.40)	0.070	-3.08 (-7.61–1.45)	0.183	4.85 (-0.99–10.69)	0.103
VFA	275	0.13 (-0.33–0.59)	0.573	-0.63 (-1.10– -0.16)	0.009	0.02 (-0.38–0.42)	0.917	0.69 (-1.10– -0.29)	0.001	-0.24 (-0.77–0.30)	0.388
VFA > 100	275	-14.06 (-63.18–35.06)	0.575	-66.37 (-116.57– − 16.17)	0.010	-24.67 (-67.14–17.80)	0.255	-81.35 (-124.28– -38.42)	< 0.001	-52.95 (-109.49– 3.60)	0.066
Body fat (%)	275	-3.17 (-5.91– -0.43)	0.024	-7.70 (-10.40– -4.99)	< 0.001	-3.21 (-5.57–-0.84)	0.008	-7.61 (-9.92– -5.30)	< 0.001	-6.84 (-9.94– -3.74)	< 0.001

^a^ Being female; ^b^ having radiographic knee osteoarthritis (rKOA); ^c^ having chronic widespread pain (CWP)

*BMI* body mass index; *PPT* pressure pain thresholds; *VFA* visceral fat area

**Table 5:** Multivariate regression analyses in the whole sample of associations between the different PPTs. Controlled for age and sex

		Trapezius bilateral		Second rib		Lateral epicondyle		Knee bilateral		Gluteal bilateral	
	n	B (95 % CI)	*p*-value	B (95 % CI)	*p*-value	B (95 % CI)	*p*-value	B (95 % CI)	*p*-value	B (95 % CI)	*p*-value
Numbers of pain sites	261	-8.47 (-14.40– -2.53)	0.005	-6.40 (-12.37– -0.42)	0.036	-7.62 (-12.99– -2.25)	0.005	-8.73 (-14.02– -3.44)	0.001	-9.44 (-16.00– -2.88)	0.005
Pain group^a^	261	-72.66 (-118.70– -26.62)	0.002	-61.68 (-107.98– -15.38)	0.009	-62.65 (-104.33– -20.97)	0.003	-57.12 (-98.54– -15.74)	0.007	-76.43 (-127.42– -25.44)	0.003
BMI	277	4.43 (-0.17–9.04)	0.059	-3.75 (-8.34–0.85)	0.110	2.56 (-1.58–6.70)	0.225	-5.21 (-9.25– -1.18)	0.011	1.90 (-3.17–6.97)	0.463
VFA	275	0.27 (-0.16–0.69)	0.215	-0.45 (-0.87– -0.03)	0.036	0.14 (-0.24–0.52)	0.473	-057 (-0.94– -0.20)	0.003	-0.03 (-0.50–0.44)	0.899
VFA > 100	275	-4.49 (-49.18–40.19)	0.844	-52.79 (-96.87– -8.72)	0.019	-16.71 (-56.72–23.31)	0.413	-71.28 (-109.59– -32.97)	< 0.001	-38.17 (-86.99–10.66)	0.125
Body fat (%)	275	0.59 (-2.24– 3.42)	0.683	-3.86 (-6.65– -1.09)	0.007	-0.53 (-3.07–-2.01)	0.682	-4.96 (-7.38– -2.54)	< 0.001	-1.96 (-5.06– -1.14)	0.215

^a^ Having chronic widespread pain (CWP)

*BMI* body mass index; *PPT* pressure pain thresholds; *VFA* visceral fat area

## Discussion

In this study of individuals with knee pain, there were no differences in pain sensitivity – as measured by PPT – between those with rKOA and no rKOA. On the other hand, pain sensitivity was associated with the female sex, having CWP, more pain sites and a higher VFA and body fat percentage. The tender points second rib and the knees were most affected. Lastly, a high prevalence of CWP was reported. The study participants had more knee symptoms than a healthy population, reporting lower KOOS in all subscales [41]. The results showed a high prevalence of CWP among women and men, of 41 and 30 %, respectively. These results are consistent with previous research [14], and the prevalence is higher than in the general population [15].

In accordance with previous studies, there were great differences in PPTs between women and men, confirming a higher pain sensitivity among women [42, 43]. However, there have been reports of bias when evaluating PPTs. Factors such as cultural and socially constructed gender roles seem to impact the results; therefore, understanding pain and central sensitisation from the biopsychosocial model is advantageous [44]. For example, feelings of masculinity have been associated with PPT, where stronger levels of emotions resulted in higher PPTs and lower levels of emotions with lower PPTs [45]. Additionally, there have been reports of higher pain acceptance among women, which could contribute to the lower reported PPTs among women [46]. Women also have a higher willingness to report pain [21] which is associated with lower reported PPTs [45]. The sex of the examiner could also affect the results. Some studies have reported lower PPTs among women and men when a female examiner is present, but some have reported higher pain tolerance [45]. The results are inconsistent, but these possible psychosocial aspects could have had an impact on the results.

The results showed no significant differences in PPT between women and men who, respectively, had and did not have rKOA. These results strengthen the suggestion that lower PPTs is not significant associated with radiographic changes [11, 12] or the severity of radiographic changes [10]. Contrary, a review by Soukas et al. [6] found lower PPT among individuals with KOA (clinical or radiographic), and Moss et al. [47] showed that individuals with clinical KOA had increased pain sensitivity and widespread hyperalgesia. The association between pain sensitivity and KOA (regardless of severity) remains unclear, and future longitudinal studies are needed.

More pain sites and having CWP were associated with higher pain sensitivity at all tender points in the univariate and multivariate regression analysis. These results were expected and indicated peripheral and central sensitisation, which in turn causes increased sensitivity [5, 48]. Associations between lower PPTs, pain intensity and pain distribution have been found in individuals with an onset of KOA [10]. Few studies have examined the association between widespread pain (not necessarily chronic) and PPT. However, pain sensitivity (assessed based on a questionnaire) and widespread pain have been shown to have a positive association [49]. Thus, the spread can be associated with the severity of pain sensitisation. In the present sample, 30 % of the men reported having CWP, whereas it is surprising that the CWP group did not report lower PPTs than the NCP/CRP group. Psychosocial factors may have impacted these results, such as high feelings of masculinity resulting in higher PPTs [45] or the lower willingness to report pain compared to women [21]. However, the lack of power with few men in the analysis could likewise be the case. More extensive studies are needed to establish the associations between men with CWP and pain sensitivity.

When studying PPTs and overweight/obesity (VFA > 100) compared to normal-weighted individuals, significant differences were found at the lateral epicondyle and the knees in women. Overweight/obese men had lower PPTs at the knees. All obesity variables (VFA, VFA > 100, and body fat) were associated with lower PPTs at the second rib and the knees. These results are in some accordance with previous studies reporting differences in pain sensitivity in various anatomical locations [12, 24]. Increased subcutaneous fat around the gluteus, trapezius and epicondyle may affect the nociceptive response and could, in some cases, decreases the response to the algometer. It is also plausible that the participants have developed more or less of general allodynia. This is part of the study’s results and is also related to the results regarding the presence of CWP.

Losing weight has resulted in less pain among overweight/obese individuals with chronic pain [50], and less pain sensitivity among obese individuals with knee pain [51], and with fibromyalgia [52]. Previous research has found associations between increased body fat and increased knee pain, along with widespread pain [53]. Together with the present study results, these findings align with the theory that increased body fat is associated with lower PPTs at the knees. One possible explanation could be adipokines, which have been found to have a lowering effect on PPTs [54] and an overall association with pain [55], especially in women [53, 56].

Future longitudinal studies are needed to understand the impact of overweight/obesity on pain sensitivity and whether increased VFA and body fat percentage could be factors of importance for increased sensitivity. According to the present study’s results, the association between pain sensitivity and overweight/obesity differs between the two sexes. Therefore, future studies should consider analysing the associations for women and men separately.

The strength of this study is that pain is assessed in a sample from the population that presents with knee pain, most with no rKOA (76 %), and thus could be regarded as an early rKOA cohort. The study also has some limitations. As pain is a subjective experience, it is difficult to measure, and some participants expressed concerns during the PPT measurement about being able to distinguish between pressure and pain. Another limitation was the lack of statistical power when stratifying for sex. Because of the few men, these results should be interpreted with caution. The number of comparisons made in the study could increase the risk of rejecting the null hypothesis, and p-values should be interpreted with this in mind.

The raters who performed the PPT procedure all had previous experience and had undergone training, and at least one hour of training has resulted in good reliability [30]. The assistant line to force application on the computer interface further increases the reliability, although having more than one test leader could still be a limitation. Lastly, a cross-sectional study cannot establish conclusions regarding the direction of the associations, whereas future longitudinal studies would be beneficial.

## Conclusions

Women had lower PPTs than men at all tender points, and pain sensitivity was not associated with rKOA, either among women or men. Having a high number of pain sites and CWP were associated with increased pain sensitivity.

The modifiable factors, increased VFA, and body fat could be risk factors for increased pain sensitivity, and health promotion interventions could decrease the risk of central sensitisation and a worsening pain state. However, longitudinal studies are needed to investigate further the associations between rKOA, CWP, overweight/obesity and pain sensitivity.

## Supplementary Information

**Additional file 1.**

**Additional file 2.**

## Data Availability

The datasets used and analysed during the current study are available from the corresponding author on reasonable request.

## Abbreviations

BMI: Body mass index
CRP: Chronic regional pain
CWP: Chronic widespread pain
KOA: Knee osteoarthritis
KOOS: Knee injury and osteoarthritis outcome score
NCP: No chronic pain
PPT: Pressure pain threshold
rKOA: Radiographic knee osteoarthritis
VFA: Visceral fat area
