# Supplementary material for: Pressure pain thresholds in individuals with knee pain: a cross-sectional study
Source: BMC Musculoskelet Disord. 2021 Jun 5;22:516. doi: 10.1186/s12891-021-04408-0 (PMC8180166; doi:10.1186/s12891-021-04408-0)
Supplement: Supplementary file 1 — Additional file 1. [file 12891_2021_4408_MOESM1_ESM.docx]

Additional file 1. PPTs in the whole sample and separately for women and men. PPTs were presented as median and interquartile range (IQR).

| PPT  median kPa (IQR) | All  n = 280 | Women  n = 199 | Men  n = 81 | p-value |
| --- | --- | --- | --- | --- |
| Trapezius bilateral | 379 (267–520) | 348 (244–472) | 509 (369–709) | <0.001 |
| Second rib | 309 (229–473) | 279 (201–372) | 480 (316–703) | <0.001 |
| Lateral epicondyle | 331 (238–463) | 307 (224–403) | 441 (300–580) | <0.001 |
| Knee bilateral | 324 (223–463) | 281 (202–368) | 463 (345–586) | <0.001 |
| Gluteal bilateral | 404 (270–607) | 354 (327–496) | 619 (453–839) | <0.001 |

*PPT, pressure pain thresholds*
