# Supplementary material for: Pressure pain thresholds in individuals with knee pain: a cross-sectional study
Source: BMC Musculoskelet Disord. 2021 Jun 5;22:516. doi: 10.1186/s12891-021-04408-0 (PMC8180166; doi:10.1186/s12891-021-04408-0)
Supplement: Supplementary file 2 — Additional file 2. [file 12891_2021_4408_MOESM2_ESM.docx]

Additional file 2. PPT in the different tender points among women and men with and without rKOA. PPTs were presented as median and interquartile range (IQR).

| PPT median kPa (IQR) | Women | |  | Men | |  |
| --- | --- | --- | --- | --- | --- | --- |
|  | rKOA  n = 45 | no rKOA  n = 143 | p-value | rKOA  n = 20 | no rKOA  n = 60 | p-value |
| Trapezius bilateral | 351 (235–510) | 331 (240–449) | 0.192 | 555 (307–775) | 507 (389–694) | 0.855 |
| Second rib | 291 (205–429) | 275 (200–364) | 0.449 | 506 (307–751) | 484 (323–690) | 0.790 |
| Lateral epicondyle | 336 (241–455) | 298 (221–384) | 0.083 | 482 (270–633) | 439 (327–567) | 0.743 |
| Knee bilateral | 271 (212–350) | 282 (202–367) | 0.981 | 549 (331–775) | 453 (352–562) | 0.289 |
| Gluteal bilateral | 379 (265–517) | 334 (231–464) | 0.184 | 604 (427–825) | 634 (479–839) | 0.781 |

*PPT, pressure pain thresholds; rKOA, radiographic knee osteoarthritis*
